# Radiation dose-event relationship after intraoperative radiotherapy as a boost in patients with breast cancer

**DOI:** 10.3389/fonc.2023.1182820

**Published:** 2023-05-05

**Authors:** Yeona Cho, Jun Won Kim, Jee Suk Chang, Ji Young Kim, Sung Gwe Ahn, Soong June Bae, Joon Jeong, Ik Jae Lee

**Affiliations:** ^1^ Department of Radiation Oncology, Gangnam Severance Hospital, Yonsei University College of Medicine, Seoul, Republic of Korea; ^2^ Department of Surgery, Gangnam Severance Hospital, Yonsei University College of Medicine, Seoul, Republic of Korea; ^3^ Department of Radiation Oncology, Yonsei Cancer Center, Heavy Ion Therapy Research Institute, Yonsei University College of Medicine, Seoul, Republic of Korea

**Keywords:** breast cancer, radiotherapy, intraoperative radiation therapy (IORT), radiation toxicites, boost radiotherapy

## Abstract

**Purpose:**

Intraoperative radiotherapy (IORT) can be used as a boost in combination with external whole breast irradiation. This study reports the clinical and dosimetric factors associated with IORT-related adverse events (AE).

**Methods and materials:**

Between 2014 and 2021, 654 patients underwent IORT. A single fraction of 20 Gy was prescribed to the surface of the tumour cavity using the mobile 50-kV X-ray source. For skin dose measurement, at least four optically stimulated luminescent dosimeter (OSLD) chips were annealed and attached to the skin edge in the superior, inferior, medial, and lateral locations during IORT. Logistic regression analyses were conducted to identify factors associated with IORT-related AE.

**Results:**

With a median follow-up period of 42 months, 7 patients experienced local recurrence, resulting in a 4-year local failure-free survival rate of 97.9%. The median skin dose measured by OSLD was 3.85 Gy (range, 0.67–10.89 Gy), and a skin dose of > 6 Gy was observed in 38 patients (2%). The most common AE was seroma (90 patients, 13.8%). We also found that 25 patients (3.9%) experienced fat necrosis during follow-up, and among them, 8 patients underwent biopsy or excision to exclude local recurrence. IORT-related late skin injury occurred in 14 patients, and a skin dose > 6 Gy was significantly associated with IORT-induced skin injury (odds ratio 4.942, 95% confidence interval 1.294–18.871, p = 0.019).

**Conclusions:**

IORT was safely administered as a boost to various populations of patients with breast cancer. However, several patients may experience severe skin injuries, and for older patients with diabetes, IORT should be performed with caution.

## Introduction

Since recurrence in the tumour bed is most common after breast-conserving surgery (BCS), radiation boost to the tumour bed is the standard treatment for breast cancer to reduce local recurrence (1–3). Boost irradiation is carried out using electrons and/or photons up to a total dose of 10–16 Gy, resulting in a prolonged treatment time of approximately one week.

The extension of the treatment period can lead to an increase in overall medical costs and patient inconvenience. Recently, the concept of intraoperative radiotherapy (IORT) during BCS has been introduced using brachytherapy or dedicated mobile IORT devices that generate fast electrons or low-energy X-rays (4–7). The Targeted intraoperative radiotherapy-A (TARGIT-A) trial used low-energy X-rays (50 kV) generated from Intrabeam (Carl Zeiss Meditec AG, Oberkochen, Germany) to deliver partial breast irradiation during BCS and reported the non-inferiority of IORT compared to whole breast irradiation (WBI) using external beam radiation therapy (EBRT) in selected patients with early breast cancer. The use of IORT with a low-energy X-ray source as a boost has been reported in several retrospective studies (8, 9), and a randomised comparison of these techniques with WBI followed by EBRT boost is currently underway in the TARGIT-B trial.

The theoretical benefits of IORT are well known; it can reduce geographical misses and provide higher doses at the surface of the tumour bed. However, little is known about the toxicity of IORT in combination with EBRT and its related factors. Previous studies have reported skin effects of radiation, such as telangiectasia and erythema, and post-IORT seroma; most were tolerable. In some cases, severe fibrosis or postoperative complications requiring surgical treatment have been reported (8, 9). In particular, in patients who receive EBRT after IORT, there is a possibility that radiation-related toxicity may increase compared with that in IORT alone; therefore, it is necessary to analyse the factors related to radiotherapy.

To determine the dose-event relationship of radiation-related toxicity, at our institution, the skin dose was measured by attaching optically stimulated luminescent dosimeters (OSLD) (InLight nanoDots, Landauer Inc., Glenwood, IL, USA) to the skin from the time of treatment initiation. Therefore, this study aimed to report the effect of skin dose measured using OSLD and other radiotherapy-related factors on adverse events (AE). This may help in determining a proper candidate for IORT as a boost.

## Methods and materials

### Patients

Since August 2014, patients diagnosed with invasive carcinoma or ductal carcinoma *in situ* (DCIS) and who underwent BCS were treated with IORT as a boost at our institution. They enrolled in this prospective, observational study. The eligibility criteria were as follows: 1) ≥ 20 years of age, 2) pathologically confirmed invasive carcinoma or DCIS, 3) American Joint Committee on Cancer stage 0–III, and 4) no previous history of chest radiation. IORT was performed concurrently with a lumpectomy.

The Institutional Review Board (IRB) approved the trial according to local laws and regulations (IRB No. 3-2017-0033). All patients provided written informed consent, and the trial was conducted in compliance with the Declaration of Helsinki.

### Treatment

Experienced surgeons at our institution conducted BCS. During BCS, a single fraction of 20 Gy was prescribed to the surface of the tumour cavity using the mobile 50-kV X-ray source (Intrabeam, Carl Zeiss, Germany). Immediately after tumour excision, frozen sections in four directions (superior, inferior, lateral, and medial) were sent to the Department of Pathology for analysis to confirm the negative margin. Re-excision was performed in cases with positive resection margins on frozen tissue examination.

A spherical applicator with an appropriate diameter (ranging from 1.5 to 5.0 cm in 0.5 cm increments) was selected according to the size of the tumour cavity, and the applicator was attached to the probe of the X-ray source. The applicator was placed inside the tumour cavity, and a purse-string suture was used to pull the walls of the tumour cavity to contact the applicator surface. The edges of the skin incision were everted so that any part of the skin was at least 1 cm away from the applicator surface to avoid excessive radiation exposure. The actual beam-on time after radiation site shielding was 20–30 min depending on the applicator diameter. The surface of the tumour cavity received 20 Gy, whereas the radiation dose was attenuated to approximately 5 Gy at a depth of 1 cm.

For each skin dose measurement, at least four OSLD chips were annealed and attached to the skin edge at the superior, inferior, medial, and lateral locations during IORT. After IORT, an InLight MicroStar reader (Landauer Inc.) was used to analyse the results of *in vivo* dosimetry. Each OSLD chip was measured three times, and the average photomultiplier tube count was recorded (**Supplementary Figure A1**).

EBRT (46 Gy in 23 fractions for WBI) was delivered 4–6 weeks after BCS + IORT or adjuvant chemotherapy. For pathological nodal involvement, regional node irradiation (RNI) was also performed; in this case, intensity-modulated radiotherapy (IMRT) was used with a total dose of 50.4 Gy in 28 fractions. Since 2019, most WBIs have been delivered with hypofractionation (40 Gy in 15 fractions) using IMRT.

### Toxicity assessment

We assessed IORT-boost-related AEs with long-term follow-up. The prespecified IORT-related AEs used in this study were as follows: 1) continued seroma aspiration 6 months after IORT or aspiration of ≥ 10 cc within 6 months, 2) haematoma requiring surgical evacuation, 3) skin breakdown or delayed wound healing, 4) Radiation Therapy Oncology Group (RTOG version 2.0) toxicity grade ≥ 3 dermatitis, and 5) any complications requiring admission. We also investigated clinically significant fat necrosis, which was accompanied by symptoms or confused with recurrence.

### Statistical analysis

The primary endpoint was IORT-related AE. A multiple logistic regression model was used to identify the factors affecting IORT-related AE. Significant factors in the univariate analysis and those that might be associated with IORT-related AE were entered into the multivariate model. Statistical significance was set at P ≤ 0.05. Local failure-free survival (LFFS) was also assessed. The survival time was calculated from the IORT dates. LFFS was estimated using the Kaplan–Meier method. These statistical analyses were performed using the commercially available statistical software SPSS version 25 (SPSS Inc., Chicago, USA). Also, for radiation dose – skin toxicity, logistic regression analysis was performed using R (version 4.1.1.)

## Results

### Patient and treatment characteristics

Between August 2014 and August 2021, 654 patients underwent IORT during BCS, followed by WBI. The median age of all cohorts was 52 years (range, 27–87 years), and the median body mass index (BMI) was 23.5 kg/m^2^ (range, 17.0–35.2). Thirty-three patients had diabetes mellitus. The majority of patients had invasive ductal carcinoma (n=513, 78.4%), followed by DCIS (n=75, 11.5%). None of the patients had tumours larger than 5 cm: T1 in 428 patients (65.4%) and T2 in 110 patients (16.8%). Most patients showed no lymph node (LN) metastasis (N = 517, 79.1%); however, two patients had pathologic N3 stage (≥ 10 LN metastasis), resulting in two patients with stage IIIC disease. Most patients (n=530, 81%) had stage I or II disease; 364 patients were in stage I (55.7%) and 166 in stage II (25.4%). More than half of the patients were luminal A-type (n=373, 57%), followed by 130 patients with luminal B, and 41 patients with human epidermal growth factor receptor-2 (HER2)-overexpression type; 16.7% showed a triple-negative subtype. More details on patient characteristics are provided in **Table 1**.

**Table 1:** Patients characteristics.

		Patients	%
		N=654	
Age	median	52	(27-87)
	< 40	43	6.6
	40-49	232	35.5
	50-59	251	38.4
	60-69	90	13.8
	≥ 70	38	5.8
Body mass index	median	23.5	(17.0-35.2)
	≥ 25	211	32.3
Diabetes	No	621	95.0
	Yes	33	5.0
Tumor type	invasive ductal	513	78.4
	invasive lobular	22	3.4
	*in situ*	75	11.5
	others	44	6.7
pathologic T stage	Tis	79	12.1
	T1	428	65.4
	T2	110	16.8
	pCR	37	5.7
pathologic N stage	N0	517	79.1
	N1	92	14.1
	N2	8	1.2
	N3	2	0.3
	NA	35	5.4
stage	0	78	11.9
	IA	362	55.4
	IB	2	0.3
	IIA	140	21.4
	IIB	26	4.0
	IIIA	8	1.2
	IIIB	0	0.0
	IIIC	2	0.3
	pCR	36	5.5
Molecular subtype	Luminal A	373	57.0
	Luminal B	130	19.9
	HER2 overexpression	41	6.3
	Triple negative	109	16.7
	NA	1	0.2
Nuclear grade	Low	16	2.4
	Intermediate	421	64.4
	High	182	27.8
	NA	35	5.4
Histologic grade	1	105	16.1
	2	345	52.8
	3	94	14.4
	NA	110	16.8
Lympho vascular invasion	negative	467	71.4
	positive	110	16.8
	NA	77	11.8
Perineural invasion	negative	556	85.0
	positive	21	3.2
	NA	77	11.8
ER	negative	150	22.9
	positive	503	76.9
	NA	1	0.2
PR	negative	231	35.3
	positive	421	64.4
	NA	2	0.3
HER2	negative	504	77.1
	positive	84	12.8
	equivocal	64	9.8
	NA	2	0.3

CR, complete remission; NA, not-assessed; ER, estrogen receptor; PR, progesterone receptor.

Treatment characteristics for all patients are shown in **Table 2**. The commonly used applicators were 3- and 3.5-cm ones (33.6% and 31.7%, respectively). Among the 654 patients, 14 did not receive EBRT, 4 were converted to total mastectomy because of repeated margin involvement, and 4 refused to receive WBI. Four patients were lost to follow-up after surgery. One patient (age: 78 years) could not receive EBRT because of wound dehiscence. The other patient showed lobular carcinoma *in situ* on permanent pathology and did not receive WBI.

**Table 2:** Treatment characteristics.

		Patients	%
		N=654	
Size of applicator	2 cm	8	1.2
	2.5 cm	111	17.0
	3 cm	220	33.6
	3.5 cm	207	31.7
	4 cm	85	13.0
	4.5 cm	22	3.4
	5 cm	1	0.2
Skin dose (Gy)	median (range)	3.85 Gy	(0.67-10.89)
	> 6 Gy	38	5.8
External beam RT	No	14	2.1
	Yes	640	97.9
Fractions	Conventional	514	78.6
	Hypofractionation	126	19.3
Regional node irradiation	No	527	80.6
	Yes	112	17.1
RT Modality	3D-CRT	406	62.1
	IMRT	234	35.8
Chemotherapy	no	389	59.5
	Neoadjuvant	91	13.9
	Adjuvant	174	26.6
Hormonal Treatment	No	145	22.2
	Yes	509	77.8

RT, radiotherapy; 3D-CRT, 3 dimensional- conformal radiotherapy; IMRT, intensity modulated radiotherapy.

Most patients underwent conventional fractionation (n=514, 78.6%), and RNI was performed in 112 patients (17.1%). Hypofractionation was used in 19.3% of patients (n=126); a comparison of patient and tumour characteristics of conventional and hypofractionation regimens is described in **Supplementary Table 1**. Ninety-one patients (13.9%) received neoadjuvant chemotherapy and 174 (26.6%) received adjuvant chemotherapy. Hormonal treatment was administered to 77.8% of patients (n=509).

### Treatment outcome

**Figure 1:**
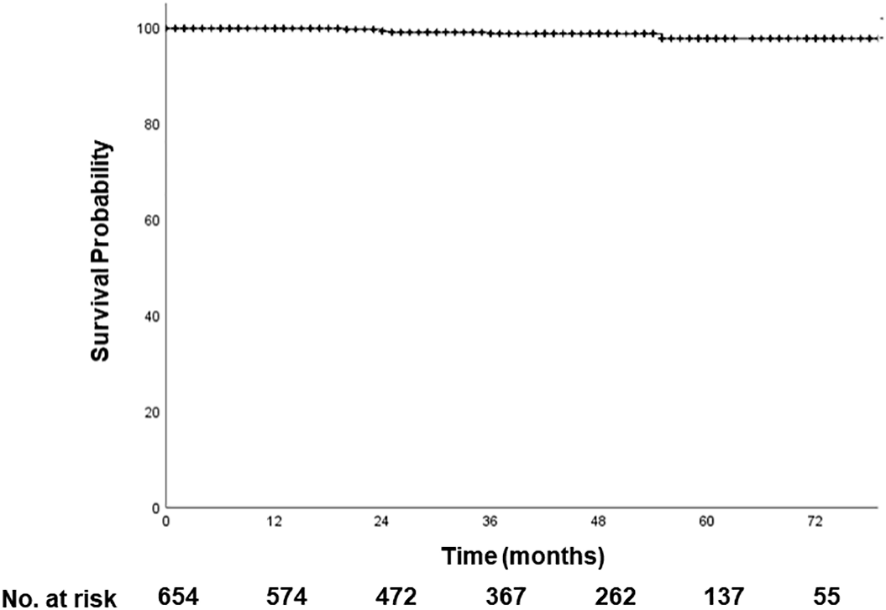
Local failure-free survival. With a median follow-up period of 42 months, 4-year LFFS is 98.9%. LFFS, local failure-free survival.

With a median follow-up period of 42 months, seven patients experienced local recurrence, resulting in a 4-year LFFS of 97.9% (**Figure 1**). Only two patients developed regional recurrence, and five developed distant metastases; the majority of the metastatic sites were mediastinal LN. No patient died. For patients who received neoadjuvant chemotherapy, the median follow-up time was 19 months (range, 2–79 months). None of these patients experienced local or regional recurrence, and two patients developed distant metastases.

### IORT-related adverse events

In total, 109 patients experienced IORT-related AE (**Table 3**). The most common AE was seroma (90 patients, 13.8%). Evacuation of the haematoma was performed in 2 patients (0.3%). Four patients were hospitalised because of infection (0.6%).

**Table 3:** Intraoperative radiotherapy-related -related adverse events.

	No of cases	(%)	Intervals
Seroma	90	14.1	median 18 months (0-72)
Hematoma	2	0.3	
Infection	4	0.6	
Wound dehiscence	11	1.7	median 18 months (3-40)
RTOG skin toxicity (≥ G3)	4	0.6	
Fat necrosis	25	3.9	

RTOG, radiation therapy oncology group.

**Figure 2:**
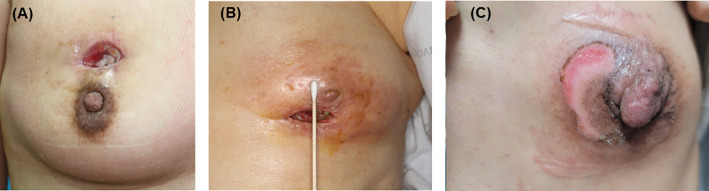
IORT-induced skin injury. **(A, B)** Wound dehiscence; **(C)** Grade 3 radiation dermatitis. IORT, intraoperative radiotherapy.

IORT-related late skin injury occurred in 14 patients (**Figure 2**); wound dehiscence, in 11 patients (1.7%); and grade 3 or higher dermatitis, in 4 patients (0.6%); with an overlap of one patient. Among them, seven patients underwent additional surgery, five underwent debridement and primary repair, and two required a local flap.

We also observed that 25 patients (3.9%) experienced fat necrosis during follow-up; among them, 8 patients underwent biopsy or excision to exclude local recurrence. Overall, clinically significant toxicities, except for seroma and fat necrosis, were observed in 21 patients (3.3%).

### Factors associated with adverse events

We analyzed the factors that could be associated with AEs (**Table 4**). Older age (> 70 years), BMI > 25 kg/m^2^, a large applicator (≥ 4 cm in diameter), and adjuvant chemotherapy were associated with increased incidence of seroma in univariate logistic regression analysis, whereas hormonal treatment was associated with a decrease in the incidence. In multivariate analysis, a large applicator was significantly associated with the development of a seroma (odds ratio [OR] 2.484, 95% confidence interval [CI] 1.443–4.276, p = 0.001), and hormonal treatment was significantly associated with decreased incidence of seroma (OR 0.528, 95% CI 0.3–0.929, p = 0.027).

**Table 4:** Factors associated with intraoperative radiotherapy-related adverse events.

	Seroma						fat necrosis						Skin injury					
	n=90						n=25						n=14					
	UA			MA														
	OR	95% CI	p	OR	95% CI	p	OR	95% CI	p	OR	95% CI	p	OR	95% CI	p	OR	95% CI	p
Age ≥ 70	1.027	1.005-1.05	0.017	2.277	0.994-5.217	0.058	1.036	0.997-1.076	0.067	1.776	0.396-7.966	0.453	5.073	1.36-18.944	0.016	3.94	0.923-16.823	0.064
BMI ≥ 25	1.985	1.261-3.125	0.003	1.404	0.856-2.303	0.179	0.841	0.365-1.936	0.684				1.866	0.667-5.215	0.234			
Diabetes	1.44	0.577-3.594	0.434				1.286	0.169-9.812	0.808				5.075	1.36-18.944	0.016	3.5	0.827-14.815	0.089
Applicator ≥ 4cm	2.973	1.798-4.917	< 0.001	2.484	1.443-4.276	0.001	0.962	0.323-2.859	0.944				2.602	0.871-7.77	0.087			
skin dose ≥ 6 Gy	0.96	0.363-2.527	0.933				0.999	0	0				4.314	1.164-15.993	0.029	4.942	1.294-18.871	0.019
IMRT	0.935	0.584-1.497	0.779				0.319	0.108-0.94	0.038	0.317	0.107-0.934	0.037	0.963	0.319-2.909	0.947			
Hypofractionation	1.145	0.661-1.985	0.629				0		0.996				0.675	0.149-3.054	0.675			
RNI	0.725	0.38-1.382	0.328				0.919	0.309-2.731	0.879				1.216	0.337-4.38	0.765			
Chemo Tx (vs none)																		
Neoadjuvant	1.722	0.905-3.276	0.098	1.061	0.501-2.246	0.876	0	0	0.997				1.434	0.285-7.226	0.662			
Adjuvant	2.19	1.288-3.485	0.003	1.573	0.901-2.748	0.111	1.808	0.803-4.067	0.152				2.676	0.886-8.082	0.081			
Hornonal Tx	0.473	0.292-0.766	0.002	0.528	0.3-0.929	0.027	1.145	0.422-3.106	0.79				1.143	0.318-4.106	0.838			

BMI, body mass index; IMRT, intensity modulated radiotherapy; RNI, regional node irradiation, Tx, treatment.

**Figure 3:**
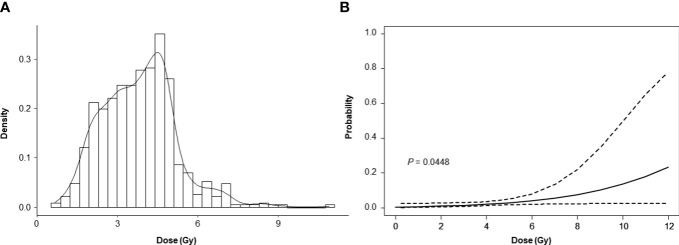
Measured skin dose **(A)** and dose – skin toxicity relationship **(B)**.

The distribution of skin dose measured by OSLD is shown in **Figure 3A**. The median skin dose was 3.85 Gy (range, 0.67–10.89 Gy), and a skin dose of > 6 Gy was observed in 38 patients (2%). The logistic regression analysis was employed to estimate the probability of developing skin toxicity according to the skin dose (**Figure 3B**). The graph shows that there is an increase in the curve starting at 6 Gy of radiation dose.

IORT-induced skin injury was associated with age > 70 years, diabetes, and a skin dose > 6 Gy. Factors related to EBRT, including IMRT, hypofractionation, and regional node irradiation, were not associated with skin breakdown. Skin doses > 6 Gy were significantly associated with IORT-induced skin injury (OR 4.942, 95% CI 1.294–18.871, p = 0.019). Old age and diabetes were associated with an only trend toward increased risk of skin injury.

We also assessed factors related to fat necrosis. Only IMRT was significantly associated with a reduced occurrence of fat necrosis (OR 0.317, 95% CI 0.107–0.934, p = 0.037).

## Discussion

In a toxicity analysis conducted with a large number of patients at a single institution, the use of IORT as a boost was found to be a safe procedure. In a previous study using IORT alone (TARGIT-A trial), all major toxicity rates were 3.3% in the IORT group and 3.9% in the EBRT group; our results are comparable to these reports despite the addition of EBRT to IORT. We demonstrated that a skin dose of > 6 Gy during IORT was significantly associated with skin injury, including wound dehiscence and RTOG grade 3 dermatitis.

This study reported a high rate of seroma aspiration (14.1%). The surgeons at our institution frequently aspirated seroma; thus, there were many cases of seroma aspiration with minimal volume (< 5 cc) or no symptoms. In the breast, seroma may occur after BCS or mastectomy. Depending on the size of the cavity, its reported occurrence varies from 15 to 57% of cases (10, 11). Patients treated with IORT have an increased risk of developing postoperative seroma (12), and the addition of EBRT could also increase this risk (13). Seroma is considered a non-serious condition; however, it may lead to substantial morbidities, such as wound failure, sepsis, prolonged wound healing, and delays in subsequent adjuvant therapy (14). The development of seroma is related to a large surgical cavity, and in this study, it was significantly associated with the size of the applicator. Therefore, for patients who are expected to have a large cavity, it would be beneficial to explain the possible complication of the occurrence of seroma.

Fat necrosis occurs in patients undergoing IORT and was less frequently reported in this study than that in the previous literature (2–52%) (15). We retrospectively evaluated fat necrosis using medical records data. Therefore, the report of fat necrosis was dependent on the physician’s experience and/or interpretation, which may have been underestimated. Fat necrosis after breast radiotherapy is a relatively common AE, and it most commonly occurs after breast brachytherapy. Generally, it is asymptomatic and has no significant clinical relevance in most cases; invasive treatment is rarely required. However, follow-up imaging study may be affected, resulting in additional diagnostic procedures. We also performed additional biopsies in eight patients among who showed fat necrosis. The evaluation of radiation factors associated with fat necrosis is limited. Wazer et al. reported that the volume of brachytherapy implants and “hotspots” was significantly correlated with the incidence of fat necrosis (16, 17). Another study showed that breast volume and V45 (breast volume receiving a radiation dose of ≥45 Gy)are associated with fat necrosis after brachytherapy (18). This study reported that IMRT could reduce the incidence of fat necrosis, probably due to homogenous dose coverage compared to three-dimensional conformal radiation therapy. Further studies are required to determine whether IMRT can reduce the risk of fat necrosis.

We showed favourable local control of IORT as a boost in a heterogeneous group of patients. Patients who had received neoadjuvant chemotherapy were included in this study. Since these patients have been treated recently, a longer follow-up period is required to determine the oncologic outcomes of IORT after neoadjuvant chemotherapy. A previous study compared the treatment outcomes of IORT and EBRT as a boost for patients treated with neoadjuvant therapy and lumpectomy (19). They reported a more favourable outcome in the IORT group with a median follow-up of 49 months. A further report with patients who had received neoadjuvant chemotherapy of our institution can support this conclusion.

Although the frequency of severe skin toxicity is low, the quality of life of these patients can be reduced because additional repair surgery and/or frequent visits are required. Higher radiation doses to the skin, old age, and diabetes are associated with severe skin injuries. A previous study revealed that preoperative breast volume, the distance between the skin and the tumour on preoperative images, and the ratio of breast volume to applicator diameter were significantly associated with skin dose (20). The use of a larger applicator in a small breast is the most significant factor for the prediction of skin dose during IORT. Therefore, IORT as a boost may not be a good choice for patients with diabetes aged over 70 years and for those with small breast volumes.

Intraoperative radiotherapy with electrons (IOERT) is another type of intraoperative radiotherapy that delivers radiation directly to the tumor bed during surgery (21). Unlike Intrabeam IORT, which uses low-energy X-rays, IOERT uses high-energy electrons to deliver radiation. IOERT is especially useful in cases where the tumor is close to critical organs or structures that could be damaged by radiation, as the electron beam can be more precisely targeted than with other types of radiation therapy. IOERT boost could be an alternative option regarding skin toxicity (22, 23). However, IOERT may require more specialized equipment and expertise than Intrabeam IORT, and may not be available at all treatment centers (24).

In addition to IORT, several other methods can reduce geographic misses. Radiation planning based on computed tomography simulation can be helpful; in particular, it is possible to accurately identify the tumour cavity through magnetic resonance-guided radiotherapy. In addition, a short course of radiotherapy, such as fast forward, can be performed in selected patients (25), Efforts have also been made to reduce the treatment duration through simultaneous integrated boost-IMRT (26, 27). Among the various possible treatment options, it is essential to comprehensively consider the patient’s age, comorbidities, and the size of the breast and tumour, in addition to its stage.

There were several problems in implementing IORT. Although our patients were enrolled in a prospective trial, in some cases, IORT proceeded without confirmation of a negative margin in the frozen section, which is a violation. In addition, others required re-excision due to a positive margin in the final pathology report after IORT; some of them underwent total mastectomy. This shows the limitation of IORT that progresses without final pathology confirmation. As IORT was used as a boost in this study, the effect of under- or overtreatment did not markedly affect patient outcomes. However, for patients treated with IORT alone, this limitation should be carefully assessed before proceeding.

This study had several limitations. First, the follow-up period was short for reporting local control of IORT as a boost. Second, the stage and treatment, except for IORT, of patients were heterogeneous (DCIS to stage III disease). Thus, additional analysis is needed for each stage after further follow-up to determine the treatment outcomes. Third, several data points, such as fat necrosis, were collected retrospectively. Therefore, there is a possibility of under- or overestimation; hence, caution is required in interpretation. Nevertheless, this study consistently measured the skin dose in all participants and, to the best of our knowledge, is the first study to evaluate the direct relationship between the skin dose and skin toxicity in patients treated with IORT. In particular, since treatment planning is not available in IORT, skin dose measurement is essential to predict severe toxicity, and we confirmed the feasibility and effectiveness of OSLD.

In conclusion, IORT as a boost was safely performed in various populations of patients with breast cancer. However, several patients may experience severe skin injuries. For older patients with diabetes, IORT should be performed with caution. Further follow-up is needed to report the local control of IORT.

## Data availability statement

The data analyzed in this study is subject to the following licenses/restrictions: Research data are stored in an institutional repository. Collaborations using this data may be possible after execution of a data-sharing agreement. Requests to access these datasets should be directed to IL, ikjae412@yuhs.ac.

## Ethics statement

The studies involving human participants were reviewed and approved by Institutional Review Board of Gangnam Severance Hospital. The patients/participants provided their written informed consent to participate in this study.

## Author contributions

Conceptualization, IL and JJ. Data curation, YC, JWK, SA, SB, and JYK. Formal analysis, YC, JWK, and JC. Investigation, YC and JC. Methodology, YC and IL. Project administration, IL and JJ. Resources, IL, SA, and JJ. Supervision, IL. Writing—original draft, YC. Writing—review and editing, YC and JWK. All authors revised the manuscript. All authors contributed to the article and approved the submitted version.
