# Supplementary figures and images for: Radiation dose-event relationship after intraoperative radiotherapy as a boost in patients with breast cancer
Source: Front Oncol. 2023 May 5;13:1182820. doi: 10.3389/fonc.2023.1182820 (PMC10196364; doi:10.3389/fonc.2023.1182820)

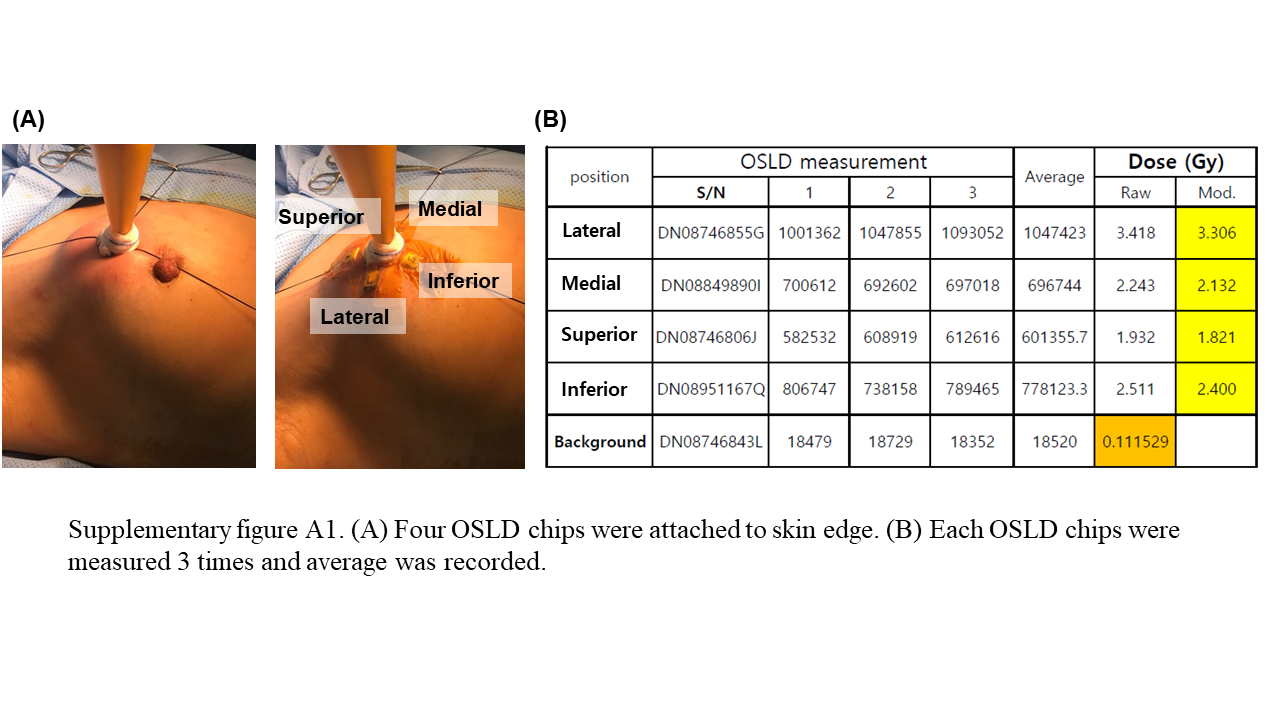

Supplement: Supplementary file 1 [file Image_1.tif]
