# Supplementary material for: Radiation dose-event relationship after intraoperative radiotherapy as a boost in patients with breast cancer
Source: Front Oncol. 2023 May 5;13:1182820. doi: 10.3389/fonc.2023.1182820 (PMC10196364; doi:10.3389/fonc.2023.1182820)
Supplement: Supplementary file 2 [file Table_1.docx]

Supplementary table 1. A comparison of patient and tumour characteristics of conventional and hypofractionation regimens

|  |  | Conventional Fx | | Hypo-Fx | |  |
| --- | --- | --- | --- | --- | --- | --- |
|  |  | N=514 | (%) | N=126 | (%) | p |
| Age | median | 52 | (27-87) | 52 | (27-81) | 0.115 |
| Body mass index | median | 23.71 | (18.2-37.49) | 23.12 | (17.02-33.02) | 0.012 |
|  | > 25 | 183 | 35.6 | 26 | 20.6 | 0.001 |
| Diabetes | No | 488 | 94.9 | 120 | 95.2 | 0.891 |
|  | Yes | 26 | 5.1 | 6 | 4.8 |  |
| Tumor type | invasive ductal | 393 | 76.5 | 110 | 87.3 | 0.016 |
|  | invasive lobular | 64 | 12.5 | 9 | 7.1 |  |
|  | in situ | 16 | 3.1 | 5 | 4.0 |  |
|  | others | 41 | 8.0 | 2 | 1.6 |  |
| pathologic T stage | Tis | 67 | 13.0 | 9 | 7.1 | 0.024 |
|  | T1 | 336 | 65.4 | 87 | 69.0 |  |
|  | T2 | 87 | 16.9 | 17 | 13.5 |  |
|  | pCR | 24 | 4.7 | 13 | 10.3 |  |
| pathologic N stage | N0 | 386 | 75.1 | 117 | 92.9 | <0.001 |
|  | N1 | 88 | 17.1 | 4 | 3.2 |  |
|  | N2 | 8 | 1.6 | 0 | 0.0 |  |
|  | N3 | 2 | 0.4 | 0 | 0.0 |  |
|  | NA | 30 | 5.8 | 5 | 4.0 |  |
| stage | 0 | 66 | 12.8 | 9 | 7.1 | 0.004 |
|  | IA | 271 | 52.7 | 86 | 68.3 |  |
|  | IB | 2 | 0.4 | 0 | 0.0 |  |
|  | IIA | 117 | 22.8 | 17 | 13.5 |  |
|  | IIB | 24 | 4.7 | 2 | 1.6 |  |
|  | IIIA | 8 | 1.6 | 0 | 0.0 |  |
|  | IIIB | 0 | 0.0 | 0 | 0.0 |  |
|  | IIIC | 2 | 0.4 | 0 | 0.0 |  |
|  | pCR | 24 | 4.7 | 12 | 9.5 |  |
| Molecular subtype | Luminal A | 299 | 58.2 | 67 | 53.2 | 0.711 |
|  | Luminal B | 97 | 18.9 | 29 | 23.0 |  |
|  | HER2 | 32 | 6.2 | 8 | 6.3 |  |
|  | Triple negative | 85 | 16.5 | 22 | 17.5 |  |
|  | NA | 1 | 0.2 | 0 | 0.0 |  |
| Nuclear grade | Low | 15 | 2.9 | 1 | 0.8 | 0.001 |
|  | Intermediate | 316 | 61.5 | 94 | 74.6 |  |
|  | High | 161 | 31.3 | 19 | 15.1 |  |
|  | NA |  | 0.0 |  | 0.0 |  |
| Histologic grade | 1 | 83 | 16.1 | 18 | 14.3 | 0.015 |
|  | 2 | 261 | 50.8 | 77 | 61.1 |  |
|  | 3 | 85 | 16.5 | 9 | 7.1 |  |
|  | NA |  | 0.0 |  | 0.0 |  |
| LVI | negative | 358 | 69.6 | 100 | 79.4 | 0.16 |
|  | positive | 91 | 17.7 | 27 | 21.4 |  |
|  | NA |  | 0.0 |  | 0.0 |  |
| PNI | negative | 434 | 84.4 | 112 | 88.9 | 0.626 |
|  | positive | 15 | 2.9 | 5 | 4.0 |  |
|  | NA |  | 0.0 |  | 0.0 |  |
| ER | negative | 117 | 22.8 | 30 | 23.8 | 0.811 |
|  | positive | 396 | 77.0 | 96 | 76.2 |  |
|  | NA |  | 0.0 |  | 0.0 |  |
| PR | negative | 183 | 35.6 | 45 | 35.7 | 0.993 |
|  | positive | 330 | 64.2 | 81 | 64.3 |  |
|  | NA |  | 0.0 |  | 0.0 |  |
| HER2 | negative | 397 | 77.2 | 97 | 77.0 | 0.876 |
|  | positive | 66 | 12.8 | 15 | 11.9 |  |
|  | equivocal | 50 | 9.7 | 14 | 11.1 |  |
|  | N/A |  | 0.0 |  | 0.0 |  |
| Neoadjuvant CTx | No | 449 | 87.4 | 100 | 79.4 | 0.021 |
|  | Yes | 65 | 12.6 | 26 | 20.6 |  |

Abbreviations: Fx, fractionation; NA, not assessed; pCR, pathologic complete remission; LVI, lymphovascular invasion; PNI, perineural invasion; ER, estrogen receptor; PR, progesterone receptor; CTx, chemotherapy
